# Effects of Cuminaldehyde on the Structural, Mechanical, UV-Barrier, and Functional Properties of Gelatin/Konjac Glucomannan Edible Films

**DOI:** 10.3390/polym18182246

**Published:** 2026-09-15

**Authors:** Xinyun Mai, Surapon Saensouk, Piyaporn Saensouk, Xingyu Wang, Qinjiabao Hu, Lingling He, Bin Huang, Caihua Liu

**Affiliations:** 1 University Engineering Research Center for Preservation and Comprehensive Utilization of Subtropical Characteristic Agricultural Products in Guangxi, Baise 533000, China; maixinyun@bsuc.edu.cn (X.M.); 11250713002@stu.ouc.edu.cn (Q.H.); helingling_1117@163.com (L.H.); liucaihua519@126.com (C.L.); 2 Guangxi Key Laboratory of Biology for Mango, College of Agriculture and Food Engineering, Baise University, Baise 533000, China; akiramak@gmail.com; 3 Office of Scientific Research, Hezhou Vocational and Technical College, Hezhou 542800, China; 4 Diversity of Family Zingiberaceae and Vascular Plant for Its Applications Research Unit, Mahasarakham University, Maha Sarakham 44150, Thailand; surapon.s@msu.ac.th (S.S.); pcornukaempferia@yahoo.com (P.S.); 5 Walai Rukhavej Botanical Research Institute, Mahasarakham University, Maha Sarakham 44150, Thailand; 6 Department of Biology, Faculty of Science, Mahasarakham University, Maha Sarakham 44150, Thailand

**Keywords:** cuminaldehyde, edible active film, gelatin, konjac glucomannan, property, structure

## Abstract

Cuminaldehyde (CA), a major bioactive constituent of cumin essential oil, was incorporated into gelatin/konjac glucomannan (Gel/KGM) composite films at different levels, expressed relative to the polymer content (mL/g polymer), to investigate its effects on film structure, physicochemical properties, and antioxidant activity. Structural characteristics of the films were examined using Fourier transform infrared spectroscopy (FTIR), Raman spectroscopy, X-ray diffraction (XRD), and scanning electron microscopy (SEM). Mechanical properties, surface wettability, optical properties, water sensitivity, and antioxidant activity were also determined. Spectroscopic analyses indicated changes in the molecular interactions of the Gel/KGM matrix following CA incorporation, while XRD and SEM analyses showed reduced structural order and a more heterogeneous microstructure at higher CA levels. Representative contact-angle observations showed an initial angle of 40.2° for the Control and 58.0° for CA-10, suggesting a tendency toward reduced surface wettability after CA incorporation. The addition of CA also improved UV-shielding properties and enhanced antioxidant responses, although the magnitude and trend of the antioxidant effect depended on the assay. In contrast, higher CA levels significantly reduced tensile strength and increased water solubility, whereas the tensile strength of films containing 1 mL CA did not differ significantly from that of the control (*p* > 0.05). These results demonstrate that the structural, physicochemical, and functional responses of Gel/KGM composite films varied with the nominal CA loading. Among the tested formulations, CA-3 represented a possible compromise between structural integrity and functional performance. Further studies using real food matrices are required to validate its practical applicability under actual food-packaging conditions.

## 1. Introduction

After harvest, fruits and vegetables are highly vulnerable to water loss, enzymatic browning, oxidative deterioration, and microbial growth, which rapidly reduces product quality and market value [1]. Postharvest losses during storage can reach 25–50%, underscoring a major challenge to global food security [2]. Conventional interventions, including low-temperature storage, modified-atmosphere storage, and chemical preservatives, can delay deterioration, but their application is often constrained by cost and safety concerns. As an alternative, edible films can form a continuous layer on the surface of fruits and vegetables, regulate the local gas composition (lower O_2_ and higher CO_2_) to suppress respiration, and reduce browning and oxidation by limiting oxygen diffusion and moisture evaporation. In addition, the film matrix can carry and gradually release antimicrobial and antioxidant agents to inhibit microbial growth [3]. Moreover, edible films are typically biopolymer-based and biodegradable, aligning with the current trend toward sustainable packaging [3]. Therefore, developing edible films with both structural stability and preservation functionality is of considerable research value.

Edible films/coatings are commonly based on proteins or polysaccharides, but single-component systems often show limitations in mechanical stability, water resistance, and functional performance. As a result, protein–polysaccharide composites with functional additives are often used to achieve structural compensation and functional enhancement [4]. Within this framework, gelatin (Gel)/konjac glucomannan (KGM) composite systems have been used to build relatively uniform and tunable network structures. The incorporation of KGM and process control (e.g., drying conditions and ultrasonic homogenization) can alter crystalline/phase assembly and strengthen intermolecular interactions, thereby affecting the mechanical and physicochemical properties of the resulting films [5,6]. However, the system remains dominated by hydrophilic chains and therefore readily absorbs moisture, softens, and swells under high humidity. As a result, maintaining structural stability and water resistance remains an important challenge. Moreover, strengthening the film formulation is often accompanied by trade-offs in visual properties, particularly transparency and color [7]. Given the need to balance barrier, stability, and functional performance in active edible films [8], it is worthwhile to examine how the incorporation of natural bioactive compounds influences both the structural and functional responses of Gel/KGM matrices.

In active edible films, incorporating essential oils or their active monomeric components is an effective approach to impart antimicrobial and antioxidant functions while also modulating barrier performance [9]. Cuminaldehyde (CA), a major aromatic aldehyde in cumin essential oil, has demonstrated antimicrobial and antioxidant activity [10]. As a plant-derived aldehyde, CA may contribute to network reconstruction through hydrophobic interactions, and its aldehyde group may also react with protein amino groups via Schiff-base formation, creating the possibility of chemical crosslinking [11]. However, its hydrophobicity and volatility create a dispersion–retention bottleneck in hydrophilic protein–polysaccharide matrices. During film formation and drying, active oil droplets tend to coalesce and phase-separate, generating micropores and interfacial defects that disrupt film continuity. Meanwhile, migration and volatilization of the active compound can reduce effective loading and compromise long-term functionality [12]. Therefore, hydrophobic active compounds such as CA may exhibit a dose-dependent trade-off: insufficient loading may limit functional performance, whereas high loading may promote dispersion defects, structural heterogeneity, and losses associated with migration or volatilization, thereby compromising overall film performance [13,14].

Current studies on active edible films mainly focus on antimicrobial/antioxidant performance, the degree of preservation improvement, and empirical changes in macroscopic properties such as tensile strength and water vapor barrier performance with additive loading [9]. However, clear multiscale evidence linking additive loading to molecular interactions, structural rearrangement, and macroscopic performance in protein–polysaccharide films remains scarce. In particular, in protein–polysaccharide composite systems, the contributions of hydrophobic actives to interfacial interactions and network rearrangement (e.g., hydrogen-bond competition, hydrophobic association, local plasticization, or weak crosslinking), as well as their influence on the coupling between optical appearance and water sensitivity, remain unclear within an integrated analytical framework. Accordingly, a single-variable dose-gradient design combined with multiscale characterization methods—including Fourier transform infrared spectroscopy (FTIR), Raman spectroscopy, X-ray diffraction (XRD), and scanning electron microscopy (SEM)—and mechanical, water-sensitivity, optical, and antioxidant evaluations can be used to examine the associations between structural changes and functional performance.

To address these gaps, Gel/KGM composite films were used as the base matrix, and a CA dose gradient was set as the main formulation variable under a constant preparation protocol. Multiscale characterization was used to examine changes in molecular interactions, ordered structure, and morphology associated with CA incorporation. Meanwhile, the films were systematically evaluated for mechanical properties, surface wettability, optical and UV-barrier properties, water resistance, and antioxidant capacity. This study aimed to compare the structural and functional responses of Gel/KGM composite films across the selected CA loadings and to assess whether an intermediate loading could provide a possible compromise between structural integrity and functional performance. The combined measurements were used to develop a cautious structure–property interpretation that may inform future formulation studies of active edible films for fruit and vegetable preservation.

## 2. Materials and Methods

### 2.1. Materials and Reagents

Gelatin (Bloom strength 250), konjac glucomannan (viscosity ≥ 15,000 mPa·s), cuminaldehyde (≥97%), glycerol (analytical grade, ≥99%), Tween-80 (analytical grade), and other reagents were purchased from Aladdin (Shanghai, China). The DPPH radical scavenging assay kit (Cat. No. A153-1-1) was purchased from Nanjing Jiancheng Bioengineering Institute (Nanjing, China), whereas the ABTS (Cat. No. G0142F) and FRAP total antioxidant capacity (T-AOC) assay kits (Cat. No. G0115F) were purchased from Geruisi Biotechnology (Suzhou, China).

### 2.2. Film Preparation and Experimental Design

Gel/KGM composite films were prepared according to the optimal formulation reported by Li et al. [15], and the effects of different CA loadings on film structure and performance were investigated on this basis. First, 12.0 g of Gel granules was added to 50 mL of ultrapure water and soaked for 6 h to allow swelling, and then stirred for 1 h in a 50 °C water bath (HH-8, Shanghai Lichen Bangxi Instrument Technology Co., Ltd., Shanghai, China) until fully dissolved to obtain the Gel stock solution. Meanwhile, 0.4 g of KGM was added to 50 mL of ultrapure water and mechanically stirred for 1 h to ensure full hydration, yielding the KGM stock solution. The two stock solutions were then mixed to obtain final concentrations of 12.0% Gel and 0.4% KGM in the system. Subsequently, 0.3 g glycerol was added as a plasticizer, and the mixture was stirred at 50 °C for 0.5 h to obtain the base Gel/KGM film-forming solution.

CA was then added to the base film-forming solution at 1, 3, and 10 mL, respectively. Tween-80 (1 mL) was added to all formulations, including the Control, as an emulsifier, and its amount was kept constant across all groups. The mixtures were subsequently stirred in a 50 °C water bath for 30 min to obtain Gel/KGM/CA film-forming emulsions with different CA loadings. The emulsions were then ultrasonically degassed at 35 °C for 15 min. Next, 25.0 g of each film-forming solution was poured into the PTFE mold and evenly cast, followed by drying in a forced-air oven at 40 °C (DHG-9070A, Shanghai Yiheng Scientific Instruments Co., Ltd., Shanghai, China) for 6 h to form films. The CA levels used in this study represent the nominal amounts initially added to the film-forming solutions. The residual CA content in the final films was not directly quantified. After film formation, the films were conditioned for 48 h at (25 ± 1) °C and (50 ± 5) % relative humidity, and then sealed for storage until use. The resulting films were used for subsequent structural characterization and performance testing. The complete compositions of the Gel/KGM film-forming formulations, including the relative CA levels and the amount of film-forming solution used for casting, are summarized in Table 1.

### 2.3. Physicochemical and Functional Characterization

#### 2.3.1. Optical Properties (Color, Opacity, UV–Vis Transmittance)

Color parameters (L*, a*, and b*) were measured using a colorimeter (NH310, 3nh, Shenzhen, China) under CIE standard illuminant D65, with an 8°/d illuminating/viewing geometry and a CIE 10° standard observer. Before measurement, the instrument was calibrated according to the manufacturer’s instructions. Film samples were placed on a white backing, and measurements were taken at 5 different positions on each film. A total of 3 independently prepared film samples were measured for each formulation. The results were expressed as the mean ± standard deviation. Total color difference (ΔE) was calculated from the L*, a*, and b* values according to [16]. Film opacity and UV-blocking properties were measured with a UV–Vis spectrophotometer (UV-2700, Shimadzu, Kyoto, Japan). For opacity measurement, the films were cut into 20 mm × 8 mm rectangles and attached to the inner polished side of a quartz cuvette, and absorbance was measured at 600 nm. Opacity was calculated using Equation (1) [16].

(1) $$\mathrm{Opacity}=\frac{A}{d}$$

where *A* is the absorbance (Abs) and *d* is the film thickness (mm).

For UV-blocking evaluation, films were cut into 20 mm × 8 mm rectangles and attached to the inner polished side of a quartz cuvette. An empty quartz cuvette was used as the blank reference, and transmittance was recorded over 200–380 nm [17]. Three independently prepared films were analyzed for each formulation for film opacity and UV-blocking properties (*n* = 3).

#### 2.3.2. Contact Angle

Surface wettability of the films was evaluated using a contact angle analyzer (DCAT 21, Data Physics Instruments, Filderstadt, Germany). In dynamic contact angle mode, a 2 μL droplet of deionized water was placed onto the film surface, and the change in contact angle over time was recorded. For each formulation, one film was analyzed using one droplet at one position. Contact angles were recorded at 0, 1, and 2 s after droplet deposition. At each time point, the reported contact angle was calculated as the mean of the left and right contact angles of the same droplet image. Because only one film and one droplet were examined for each formulation, the contact-angle measurements were treated as descriptive observations and were not subjected to inferential statistical analysis.

#### 2.3.3. Thickness and Tensile Strength

Film thickness was measured at different positions using a digital thickness gauge (DC 273877, Yueqing Hemu Instrument Co., Ltd., Yueqing, China). For each sample, no fewer than six points were randomly selected, and the mean value was recorded to 0.001 mm.

The tensile properties of the films were measured using a force tester (LTZ-2, Changshu Shuangjie Testing Instrument Factory, Suzhou, China). The films were cut into strips of 10 mm × 50 mm. Three independently prepared films were analyzed for each formulation, with one strip obtained from each film (*n* = 3). The tester was operated in its manual loading mode, and the specimens were pulled until rupture while the maximum force at break (F) was recorded. A programmable crosshead speed was not available, and the initial grip separation was not separately recorded. Therefore, the tensile results were used primarily for comparative evaluation among formulations under the same testing conditions. Tensile strength (TS) was calculated according to Equation (2) [18].

(2) $$\mathrm{TS}=\frac{F}{L\times d}$$

where *F* is the maximum force at film break (N), *L* is the film width (mm), and *d* is the film thickness (mm).

#### 2.3.4. Water Sensitivity (Moisture Content, Swelling Degree, Water Solubility)

Films were cut into circular discs (28 mm in diameter) and weighed (m_1_). The samples were then dried in an oven at 105 °C for 24 h to obtain the dry weight (m_2_). After drying, the films were immersed in 50 mL deionized water for 24 h, blotted with filter paper to remove surface water, and weighed again (m_3_). Finally, the films were dried again at 105 °C for 24 h until constant weight was reached (m_4_). Moisture content (MC), swelling degree (SD), and water solubility (WS) were calculated using Equations (3)–(5), respectively [19]. Three independently prepared films were analyzed for each formulation for MC, SD, and WS (*n* = 3).

(3) $$\mathrm{MC}\;(\%)=\frac{m_{1}-m_{2}}{m_{1}}\times 100$$

(4) $$\mathrm{SD}\;(\%)=\frac{m_{3}-m_{2}}{m_{2}}\times 100$$

(5) $$\mathrm{WS}\;(\%)=\frac{m_{2}-m_{4}}{m_{2}}\times 100$$

#### 2.3.5. Antioxidant Activity (DPPH, ABTS, FRAP)

Antioxidant activity of the films was evaluated using commercial DPPH (A153-1-1, Nanjing Jiancheng Bioengineering Institute, Nanjing, China), ABTS (G0142F, Geruisi Biotechnology, Suzhou, China), and FRAP (G0115F, Geruisi Biotechnology, Suzhou, China) assay kits. Equilibrated films were cut into small pieces, and approximately 0.1 g of each sample was extracted with 1.0 mL of 80% ethanol. For the DPPH and ABTS assays, the extracts were centrifuged at 12,000 rpm for 10 min, with the ABTS extraction performed at 4 °C, and the supernatants were collected for analysis. For the FRAP assay, the samples were ultrasonically extracted at 60 °C and 200–300 W for 30 min, followed by centrifugation at 12,000 rpm for 10 min. For the DPPH assay, 400 μL of sample extract was mixed with 600 μL of DPPH working solution, incubated in the dark at room temperature for 30 min, and centrifuged at 4000 rpm for 5 min before absorbance was measured at 517 nm. For the ABTS assay, 40 μL of sample extract was mixed with 760 μL of ABTS working solution and incubated at 25 °C in the dark for 6 min before measurement at 414 nm. For the FRAP assay, 75 μL of sample extract, 75 μL of distilled water, and 850 μL of freshly prepared chromogenic solution were mixed and incubated at 25 °C for 10 min before measurement at 590 nm. Antioxidant capacities were quantified using the corresponding Trolox calibration curves and expressed as μmol Trolox/g film. Three independently prepared films were analyzed for each formulation for the DPPH, ABTS, and FRAP assays (*n* = 3).

### 2.4. Structural Characterization

#### 2.4.1. Analysis of Molecular Interactions by Fourier Transform Infrared Spectroscopy (FTIR)

Fourier transform infrared spectra were collected using a Nicolet Summit X spectrometer (Thermo Fisher Scientific, Madison, WI, USA) in attenuated total reflectance (ATR) mode. The scanning range was 4000–400 cm^−1^, with a resolution of 2 cm^−1^ and 32 accumulated scans per sample. Before measurement, film samples were equilibrated under dry conditions to minimize interference from moisture in the infrared spectra.

#### 2.4.2. Molecular Vibrational Analysis by Raman Spectroscopy

Raman spectroscopy was performed using an HR Evolution spectrometer (HORIBA Scientific, Kyoto, Japan). A 785 nm laser was used as the excitation source at a laser power of 50 mW. Spectra were collected over 50–4000 cm^−1^ using a 100× microscope objective, with an integration time of 25 s and three accumulations. The spectral resolution was 2 cm^−1^.

#### 2.4.3. Crystalline Structure Analysis by X-Ray Diffraction (XRD)

The crystalline structure of the films was analyzed using an X-ray diffractometer (SmartLab 9 kW, Rigaku, Tokyo, Japan) with Cu Kα radiation (λ = 0.154056 nm). The X-ray tube was operated at 35 kV and 25 mA. Diffraction patterns were recorded over a 2θ range of 5–90° with a step size of 0.02° and a scanning rate of 2°/min.

#### 2.4.4. Microstructural Analysis by Scanning Electron Microscopy (SEM)

The surface and cross-sectional microstructures of the films were observed using a scanning electron microscope (Sigma 360, Carl Zeiss, Jena, Germany) equipped with an energy-dispersive spectrometer (Xplore 30, Oxford Instruments, High Wycombe, UK). Samples were fractured in liquid nitrogen to obtain clean, brittle cross-sectional surfaces, sputter-coated with an approximately 10 nm thick gold layer, and then observed under SEM at an accelerating voltage of 5 kV. Surface and cross-sectional images were captured at 1000× magnification. For each formulation, three independently prepared films were examined, with at least three surface fields and three cross-sectional fields evaluated for each film.

### 2.5. Spectral Data Processing and Quantitative Analysis

#### 2.5.1. Spectral Preprocessing and Feature Extraction

**Figure 1:**
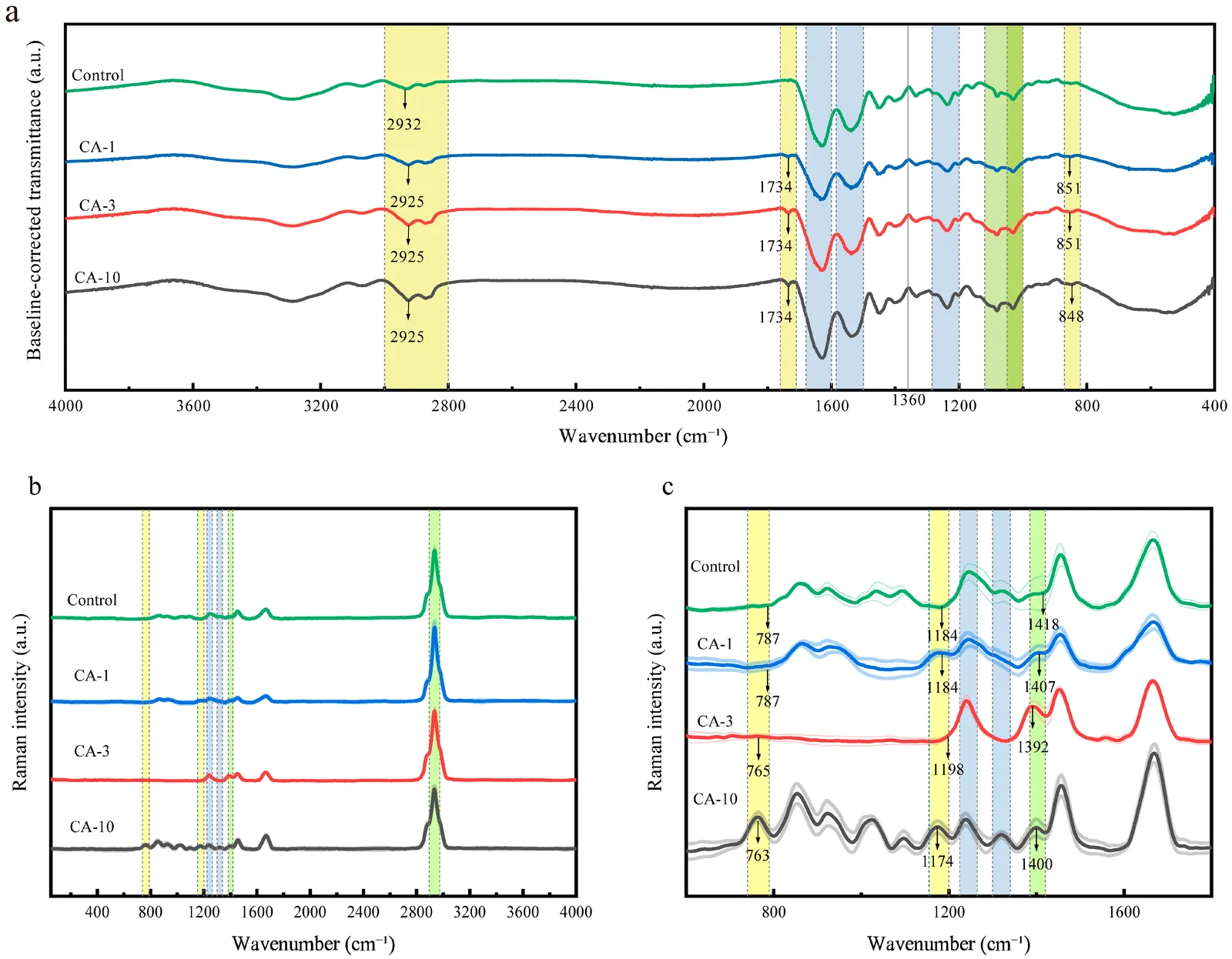
FTIR and Raman spectral evidence of CA-associated molecular interactions in films. (**a**) Baseline-corrected FTIR spectra with characteristic spectral windows and representative peak shifts; (**b**) original Raman spectra (full range) with selected analysis windows; (**c**) magnified original Raman spectra in the fingerprint region highlighting CA-responsive bands. The Raman spectra shown in (**b**,**c**) were not subjected to baseline correction or normalization and were vertically offset for clarity.

Before FTIR and Raman spectral feature extraction, baseline correction was performed using the asymmetric least squares (AsLS) method. Subsequent peak feature extraction, reference-band selection, and stability assessment were all conducted on the baseline-corrected spectra. The Raman spectra displayed in Figure 1b,c are the original spectra without baseline correction or normalization and are shown only for visualization of the spectral profiles. Based on predefined functional-group spectral windows, intensity metrics (peak depth/height and integrated area) and peak-position/band-shape metrics (peak position, center of gravity (COG), and full width at half maximum (FWHM)) were extracted to characterize CA-induced changes in absorption/scattering intensity, band-shape reconstruction, and peak shifts.

#### 2.5.2. Reference-Band Normalization and Ratio-Feature Construction

To reduce the influence of non-specific factors, including film-thickness differences, fluctuations in measurement conditions, and overall signal drift, reference-band normalization was applied and ratio-based features were constructed. The use of a stable internal reference band for FTIR normalization has been applied in polymer systems to improve spectral comparability. In the FTIR analysis, a COO^−^-related band near 1360 cm^−1^ (1340–1380 cm^−1^) was selected as the normalization reference, and the peak depth and integrated area of each functional-group window were normalized as ratios to the corresponding parameters of this reference band. For quantitative Raman feature analysis, the baseline-corrected spectra were normalized using the reference band at 2889 cm^−1^ (2840–2890 cm^−1^). Spectral preprocessing, feature extraction, reference-band screening, and stability evaluation were all performed in R (version 4.5.1), with RStudio (version 2025.5.1.513) used as the development and execution platform.

#### 2.5.3. Trend Analysis and Reference-Band Screening Metrics

To evaluate the suitability of candidate reference bands and quantify dose-dependent spectral responses, trend and stability metrics were calculated using the four CA loading levels (0, 1, 3, and 10 mL). For each metric, group means (rather than all pointwise replicates) were used for trend analysis to reduce within-group measurement noise and to reflect the formulation-level response.

For candidate reference-band screening, the following indicators were considered: peak-position standard deviation (SD), coefficient of variation (CV) of peak intensity (peak depth/height) and integrated area, Spearman’s rank correlation coefficient (Spearman’s ρ) between CA loading and the corresponding intensity metric, and the standardized slope of a linear fit. Candidate reference bands were evaluated based on the combined consideration of positional stability, intensity variation, and dose sensitivity, rather than on any single metric.

Given that CA loading included only four ordered levels and the response variables were not assumed to be linear or normally distributed, Spearman’s ρ was used. The standardized slope was calculated as follows:

(6) $$\mathrm{Standardized}\;\mathrm{slope}\;=\;\frac{\mathrm{slope}\;\mathrm{from}\;\mathrm{linear}\;\mathrm{regression}\;(y∼\mathrm{CA}\;\mathrm{loading})}{\mathrm{median}(y)}$$

where y represents the corresponding spectral metric (e.g., peak depth/height or integrated area). This normalization allows direct comparison of dose sensitivity across spectral bands with different absolute signal magnitudes.

#### 2.5.4. Statistical Analysis

Three independent film-forming preparations were performed for each formulation (*n* = 3). Unless otherwise specified, the reported values represent measurements obtained from these three independently prepared film batches. The number of technical replicates or measurement positions for each determination is specified in the corresponding methodological subsection. Results with independent experimental replication are presented as mean ± standard deviation (mean ± SD), and differences among groups were analyzed by one-way analysis of variance (one-way ANOVA) followed by Tukey’s multiple comparison test, with statistical significance set at *p* < 0.05. Contact-angle measurements, for which only one film and one droplet at one position were examined per formulation, were not included in the inferential statistical analysis and are presented descriptively.

## 3. Results and Discussion

### 3.1. CA-Induced Molecular Interactions Revealed by FTIR and Raman

#### 3.1.1. Reference-Band Validation and Normalization Strategy

To ensure the robustness of subsequent ratio-feature construction, the stability of the FTIR and Raman reference bands and their sensitivity to changes in CA addition were first systematically evaluated (Table 2). For FTIR, the 1360 cm^−1^ band (1340–1380 cm^−1^) showed no detectable peak-position shift across different CA levels (peak-position SD = 0.000 cm^−1^), and its intensity metrics responded only weakly to dosage variation: the CV values of peak depth and peak area were 0.210 and 0.201, respectively, both with Spearman’s ρ = 0.40, and the corresponding standardized slopes were 0.025 and 0.026. These results indicate that this band did not exhibit a pronounced systematic change with increasing CA loading under the present experimental conditions and is therefore suitable as an FTIR reference band for ratio normalization [20]. For Raman, the 2889 cm^−1^ band (2840–2890 cm^−1^) also showed high stability, with a peak-position SD of 1.120 cm^−1^ and CV values of 0.093 and 0.122 for peak height and peak area, respectively, indicating limited overall fluctuations in band shape and intensity. Although this band showed some correlation with CA loading (Spearman’s ρ = 0.60), the corresponding standardized slopes were low (0.008 for peak height and 0.011 for peak area), suggesting a limited response amplitude and the absence of high sensitivity. Therefore, the 2889 cm^−1^ band was selected as the reference band for whole-spectrum Raman normalization. This reference-band screening provides a unified and comparable normalization basis for the subsequent dose–response analysis of FTIR and Raman feature regions and improves the reliability of cross-technique interpretation. The trend and stability metrics used for reference-band screening (including Spearman’s ρ and standardized slope) are defined in Section 2.5.3.

After AsLS baseline correction and normalization to the 1360 cm^−1^ reference band, the FTIR feature bands exhibited markedly differentiated responses to CA addition (Table 3, Figure 1a). Based on common FTIR assignments for Gel/KGM composite systems, the O–H/N–H stretching region, aliphatic C–H stretching region, amide I/II/III regions, and the polysaccharide backbone-related C–O–C/C–O vibration regions reflect structural changes in the hydrogen-bonding environment, hydrophobic chain segments, protein backbone, and polysaccharide chains, respectively [5].

The carbonyl-related region (~1734 cm^−1^) was one of the most CA-sensitive FTIR regions. This window showed an area ratio of 9.712, a Spearman’s ρ of 0.8, and a standardized slope of 0.095 (Table 3), indicating a pronounced dose–response behavior. Both the peak position and the center of gravity (COG) shifted to higher wavenumbers (ΔPeak position = +24.106 cm^−1^; ΔCOG = +16.915 cm^−1^), and a more distinct absorption peak was observed at ~1734 cm^−1^ in the CA-treated groups (Figure 1a). These changes suggest an increase in the carbonyl-related signal and a shift in the relative contributions of vibrational components within this band after CA addition. However, this band cannot be attributed exclusively to CA, because KGM itself contains carbonyl-containing acetyl groups that may contribute to absorption in this region [21]. Moreover, changes in the local hydrogen-bonding environment within the Gel/KGM/CA matrix may affect the position and intensity of this band. Therefore, the observed changes at ~1734 cm^−1^ are more appropriately interpreted as a redistribution of carbonyl-related vibrational contributions and changes in the local molecular environment rather than as direct evidence of a CA-specific carbonyl contribution.

The aliphatic C–H stretching region (2800–3000 cm^−1^) showed a clear dose-dependent response, with an Area ratio of 2.574 and a monotonic trend (Spearman’s ρ = 1.0; Table 3). This region also exhibited pronounced band-shape variation, including downward shifts in peak position and COG (ΔPeak position = −6.991 cm^−1^; ΔCOG = −13.530 cm^−1^) and a marked increase in FWHM (ΔFWHM = +47.080 cm^−1^). This coordinated pattern of “intensification–red shift–broadening” suggests that CA addition not only increased the contribution of aliphatic C–H vibrations but also enhanced local microenvironmental heterogeneity and band overlap, consistent with rearrangement and packing-state adjustment of hydrophobic-related domains.

In the fingerprint region, polysaccharide backbone-related bands showed moderate but directionally consistent co-enhancement. The polysaccharide C–O–C/C–O vibration region (1050–1120 cm^−1^) had an Area ratio of 2.168 (Spearman’s ρ = 0.8), accompanied by a clear COG shift (ΔCOG = +6.099 cm^−1^), while the polysaccharide C–O vibration region (1000–1050 cm^−1^) showed an Area ratio of 1.411 (Spearman’s ρ = 0.8), with a largely stable peak position and intensity enhancement as the dominant change (Table 3). The coordinated changes in these two bands indicate that, after CA introduction, polysaccharide chain-related vibrations still exhibited a clear dose-dependent enhancement under normalized conditions, and that in some windows the changes were better characterized by band-shape evolution and redistribution of component contributions rather than by simple peak-position shifts. This supports the interpretation that CA introduction affects not only characteristic peak intensity but also the local structural environment surrounding the polysaccharide chains [5].

In contrast, the amide I/II/III regions showed only slight enhancement and limited changes in peak position or COG overall (Table 3), suggesting that CA caused only mild direct perturbation to the characteristic bands of the protein main backbone under the present conditions. This suggests that the primary FTIR-level effects of CA were more strongly expressed in the carbonyl, hydrophobic-chain, and polysaccharide-backbone-related vibrational regions, rather than in major reconstruction of the protein main backbone under the present conditions. Particularly, the aromatic ring-related characteristic band (~848 cm^−1^) showed only a small area-response amplitude (Area ratio = 1.113; Spearman’s ρ = 0.4), but it contained substantial discriminative information in its peak-position change. In this window, the peak position shifted markedly from near the window boundary to ~848 cm^−1^ (ΔPeak position = +27.963 cm^−1^), while the COG also moved upward (ΔCOG = +2.744 cm^−1^) (Table 3). Correspondingly, a clearer absorption feature appeared at ~848–851 cm^−1^ in the CA-treated spectra (Figure 1a). This “low-amplitude but highly informative” response suggests that the aromatic-related vibrational signal became more resolved (from a weak and unstable state) after CA addition, and it provides important cross-evidence for the subsequent joint interpretation of Raman aromatic-related windows.

Overall, the FTIR results demonstrate systematic spectral changes associated with CA addition, particularly in the carbonyl-, C–H-, and polysaccharide-related regions. These changes were characterized primarily by variations in peak position, COG, and band shape, providing a basis for comparison with the Raman spectral responses and subsequent structural analyses by XRD and SEM.

#### 3.1.2. Raman Evidence of Aromatic-Related Structural Contribution and Protein/CH-Domain Redistribution

After AsLS baseline correction and normalization to the 2889 cm^−1^ reference band, the quantitative Raman feature analysis showed differentiated responses to CA addition (Table 4), while the corresponding original spectra are shown in Figure 1b,c for visual comparison. These results provide evidence complementary to FTIR that is consistent with aromatic-structure involvement, protein-related domain rearrangement, and redistribution of CH-related domains.

Among the analyzed Raman regions, the 765 cm^−1^ (740–790 cm^−1^) and 1180 cm^−1^ (1155–1200 cm^−1^) bands showed the strongest CA-associated responses. The 765 cm^−1^ band showed an Area ratio of 7.286 and a highly consistent dose trend (Spearman’s ρ = 1.0; standardized slope = 0.567), indicating stable enhancement with increasing CA addition. Both the peak position and COG shifted to lower wavenumbers (ΔPeak position = −23.724 cm^−1^; ΔCOG = −1.700 cm^−1^), indicating that this band window showed not only increased intensity but also a shift in the relative contributions of vibrational components within the band family. The 1180 cm^−1^ band showed a larger increase at the endpoint (Area ratio = 8.775), but weaker dose monotonicity (Spearman’s ρ = 0.4; CV = 0.903), consistent with a stage-dependent or nonlinear response. Nevertheless, this region also exhibited clear low-wavenumber shifts (ΔPeak position = −10.307 cm^−1^; ΔCOG = −7.969 cm^−1^), indicating pronounced band-shape reconstruction. Taken together, the strong responses at 765/1180 cm^−1^ are consistent with an increased spectroscopic contribution from CA-related aromatic structural units and associated changes in the local vibrational environment.

In contrast to the enhancement of aromatic-related bands, the 1245 cm^−1^ (1225–1265 cm^−1^) and 1320 cm^−1^ (1300–1340 cm^−1^) bands (amide III-related region) generally showed relative weakening and rearrangement. The 1245 cm^−1^ band had an Area ratio of 0.745 and was negatively correlated with dose (Spearman’s ρ = −0.8; CV = 0.136), while both the peak position and COG shifted toward lower wavenumbers (ΔPeak position = −7.457 cm^−1^; ΔCOG = −2.114 cm^−1^). The 1320 cm^−1^ band also showed a decreasing trend (Area ratio = 0.774; Spearman’s ρ = −0.8), accompanied by a peak-position shift (ΔPeak position = −1.843 cm^−1^) (Table 4). These results suggest that, after CA addition, the relative contribution of protein-backbone-related vibrational regions decreased and the local coupling environment was adjusted, supporting the interpretation that protein-related domains were perturbed and rearranged [22].

In the CH_2_/CH_3_-related domains, the 1400 cm^−1^ (1385–1420 cm^−1^, CH_2_/CH_3_ deformation-related) and 2935 cm^−1^ (2895–2975 cm^−1^, CH_2_/CH_3_ stretching-related) bands showed coordinated but directionally different changes. The 1400 cm^−1^ band exhibited moderate enhancement (Area ratio = 1.367; Spearman’s ρ = 0.8), but with a relatively large peak-position shift (ΔPeak position = −18.223 cm^−1^; ΔCOG = −1.276 cm^−1^), indicating substantial adjustment in band-family composition in this region. In contrast, the 2935 cm^−1^ band showed a stable decrease (Area ratio = 0.855; Spearman’s ρ = −0.8; CV = 0.160), with slight downward shifts in both peak position and COG (ΔPeak position = −2.220 cm^−1^; ΔCOG = −1.225 cm^−1^). Notably, 2935 cm^−1^ was the only key response window in which a clear FWHM change was detected (ΔFWHM = +0.838 cm^−1^), suggesting increased heterogeneity or enhanced vibrational coupling in the high-wavenumber CH stretching region [23]. Changes at 1400 and 2935 cm^−1^ suggest that CA incorporation was associated with changes in the local CH-associated microenvironment and in the relative vibrational contributions of CH-related domains.

In addition, the 850 cm^−1^ band (830–875 cm^−1^), although showing limited dose monotonicity (Spearman’s ρ = 0.4), still exhibited an enhancement trend (Area ratio = 2.132), accompanied by downward shifts in both peak position and COG (ΔPeak position = −6.850 cm^−1^; ΔCOG = −1.334 cm^−1^). Given that this window lies near the aromatic-related vibrational region and corresponds well to the enhancement and clarification of the ~848 cm^−1^ aromatic ring-related feature in FTIR (Figure 1a), this Raman window serves as supplementary evidence consistent with aromatic-structure participation in local interactions within the film. In particular, the FTIR signal near ~848 cm^−1^ reflects a band-shape transition “from weak to resolvable,” while the Raman responses at 765/850/1180 cm^−1^ further strengthen the interpretation of aromatic incorporation and local environmental changes from the perspective of scattering signals.

From the perspective of FTIR–Raman cross-validation, the two techniques showed high consistency across key structural domains. The dose-dependent response of the FTIR carbonyl-related region near ~1734 cm^−1^ is consistent with changes in carbonyl-related vibrational contributions and the local molecular environment, rather than providing direct evidence of a CA-specific carbonyl contribution [21]. The enhancement, red shift, and broadening of the FTIR aliphatic C–H region are corroborated by the coordinated changes at Raman 1400/2935 cm^−1^, indicating altered local environments and chain-packing states in CH-related domains. The improved resolution of the FTIR aromatic ring-related feature near ~848 cm^−1^, together with the Raman responses at 765/850/1180 cm^−1^, is consistent with an increased spectroscopic contribution from CA aromatic structural units. Importantly, increases in band intensity alone cannot distinguish the direct contribution of CA from specific interactions with the Gel/KGM matrix. Therefore, changes in the local molecular environment are interpreted primarily from the observed peak-position shifts, COG changes, and band-shape variations rather than from intensity enhancement alone.

### 3.2. Mesoscopic Structural Evolution: Crystallinity and Morphology

#### 3.2.1. XRD: Weakening and Rearrangement of Ordered Domains

**Figure 2:**
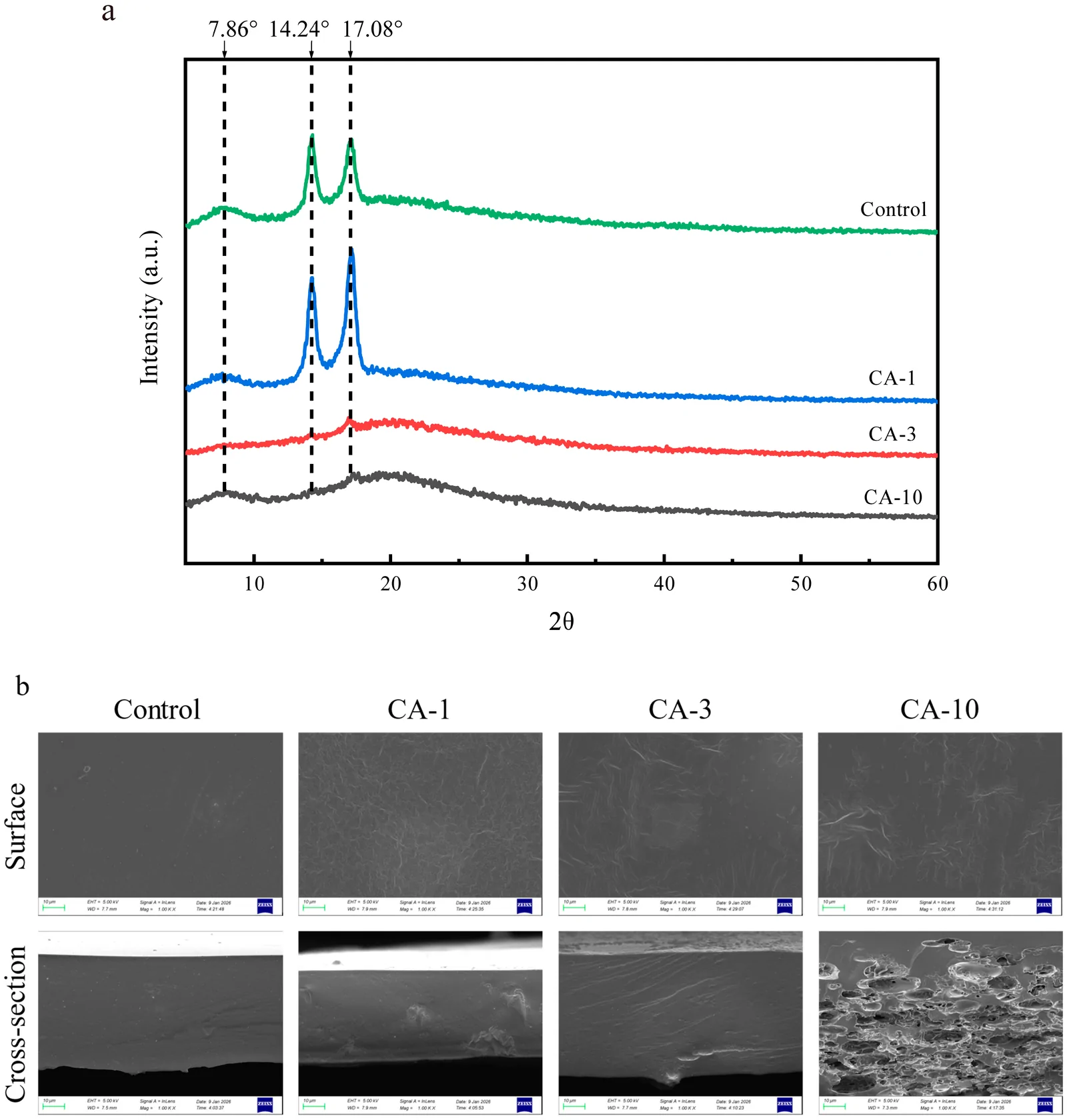
Mesostructural evolution of films as affected by CA loading: (**a**) XRD patterns showing the evolution of characteristic diffraction peaks; (**b**) SEM micrographs of film surfaces and cross-sections. The XRD patterns were not normalized and were vertically offset for clarity. All SEM micrographs were acquired at 1000× magnification with a scale bar of 10 μm.

The XRD patterns showed qualitative changes in the ordered structural features of the composite films following CA addition (Figure 2a), with a clear dose-dependent trend. The Control film showed characteristic peaks at 7.86°, 14.24°, and 17.08° in the low- and mid-angle regions, indicating that residual ordered domains were retained in the film matrix. After addition of a low CA dose (CA-1), the corresponding peaks remained detectable at 7.52°, 14.24°, and 17.12°, and only slight changes in peak profile were observed. This indicates that low-dose CA largely preserved the overall ordered framework of the composite film while causing limited rearrangement in local ordered domains.

As CA loading increased to CA-3 and CA-10, the diffraction peaks showed overall broadening, blunting, and peak-position migration (CA-3: 9.50°, 14.20°, and 17.04°; CA-10: 8.12°, 15.50°, and 17.34°). Importantly, the characteristic diffraction peaks remained detectable in CA-10, indicating that residual ordered domains were retained in the film matrix. Therefore, the observed peak broadening, blunting, and migration should not be interpreted as complete amorphization. Rather, these qualitative changes in the XRD patterns suggest weakening and rearrangement of ordered domains, together with increased amorphous characteristics [24]. Specifically, the shift and broadening of the low-angle peak suggest reduced stability of ordered domains associated with chain packing. In the 14–18° region, the peak cluster gradually changed from relatively sharp to broader and blunter features, with more pronounced peak rearrangement in the high-dose group, indicating reconstruction of ordered domains in the mid-angle region and enhanced amorphous characteristics. This coordinated pattern of “increased peak width–peak blunting–peak migration” is consistent with continuous disturbance of the regular packing mode of polymer chains after CA addition, thereby weakening the overall order of the system [25].

These results connect well with the preceding spectroscopic evidence: the local interaction changes in carbonyl, aromatic, and CH-related domains revealed by FTIR/Raman were further manifested at the XRD level as weakening and rearrangement of mesoscopic ordered domains, suggesting that CA likely exerts a continuous influence on the composite film from the molecular vibrational level to the ordered-structure level. Thus, the qualitative XRD patterns suggest that increasing CA loading reduced the degree of structural order without completely eliminating the residual ordered domains within the Gel/KGM matrix.

#### 3.2.2. SEM: Surface Heterogeneity and Cross-Sectional Morphology

The SEM results further supported CA-associated structural reconstruction at the morphological scale (Figure 2b). In terms of surface morphology, the Control film was generally smooth and dense, with only a few fine particles or local irregular textures. After low-dose CA addition (CA-1), relatively uniform fine wrinkles and fibrous textures appeared on the film surface, while overall continuity remained good, indicating that CA had already induced surface microstructural rearrangement but had not yet caused obvious structural damage. As CA loading increased to CA-3, banded and oriented wrinkle features became more pronounced, and the local surface texture appeared more irregular. The CA-10 group showed the most heterogeneous surface morphology, with coarser wrinkle networks and more nonuniform structural regions [25,26].

The evolution of cross-sectional morphology was consistent with the surface trend and more directly reflected changes in internal structural continuity. The Control film exhibited a dense and continuous cross-section with smooth interfaces, showing a typical uniform compact structure. The CA-1 film still maintained good overall continuity, with only slight undulations and local micro-defects, suggesting that low-dose CA had a relatively mild effect on the internal film skeleton. In the CA-3 group, the cross-section began to show layered structures and enhanced local undulation, indicating adjustments in chain-packing mode and a transition of the internal film structure from uniformly compact to locally rearranged. In contrast, the CA-10 group exhibited a honeycomb-like cross-sectional morphology characterized by more frequent voids and less compact regions. These qualitative morphological observations suggest reduced internal continuity and compactness of the film matrix and may indicate a greater tendency toward phase separation or structural heterogeneity at high CA loading [27]. It should also be noted that Tween-80 was maintained at a fixed amount (1 mL) across all formulations; therefore, the Tween-80/CA ratio decreased with increasing CA loading. This variation may have influenced CA dispersion and interfacial organization, particularly at higher CA levels, and may have partly contributed to the observed structural heterogeneity and phase-separation tendency. Accordingly, the dose-dependent morphological changes should be interpreted as resulting primarily from increasing CA loading, while also considering the potential contribution of the varying Tween-80/CA ratio. In addition, CA is a volatile compound, and some loss may occur during film preparation and drying [14]. Since the residual CA content was not directly quantified in the dried films, the actual amount of CA retained in the film matrix remains uncertain. Therefore, the dose-dependent structural changes observed in this study should be interpreted in relation to the nominal CA loading rather than the residual CA concentration in the final films.

### 3.3. Effect of Cuminaldehyde on the Physicochemical and Functional Properties of Gelatin/Konjac Glucomannan Edible Films

#### 3.3.1. Optical Properties

**Figure 3:**
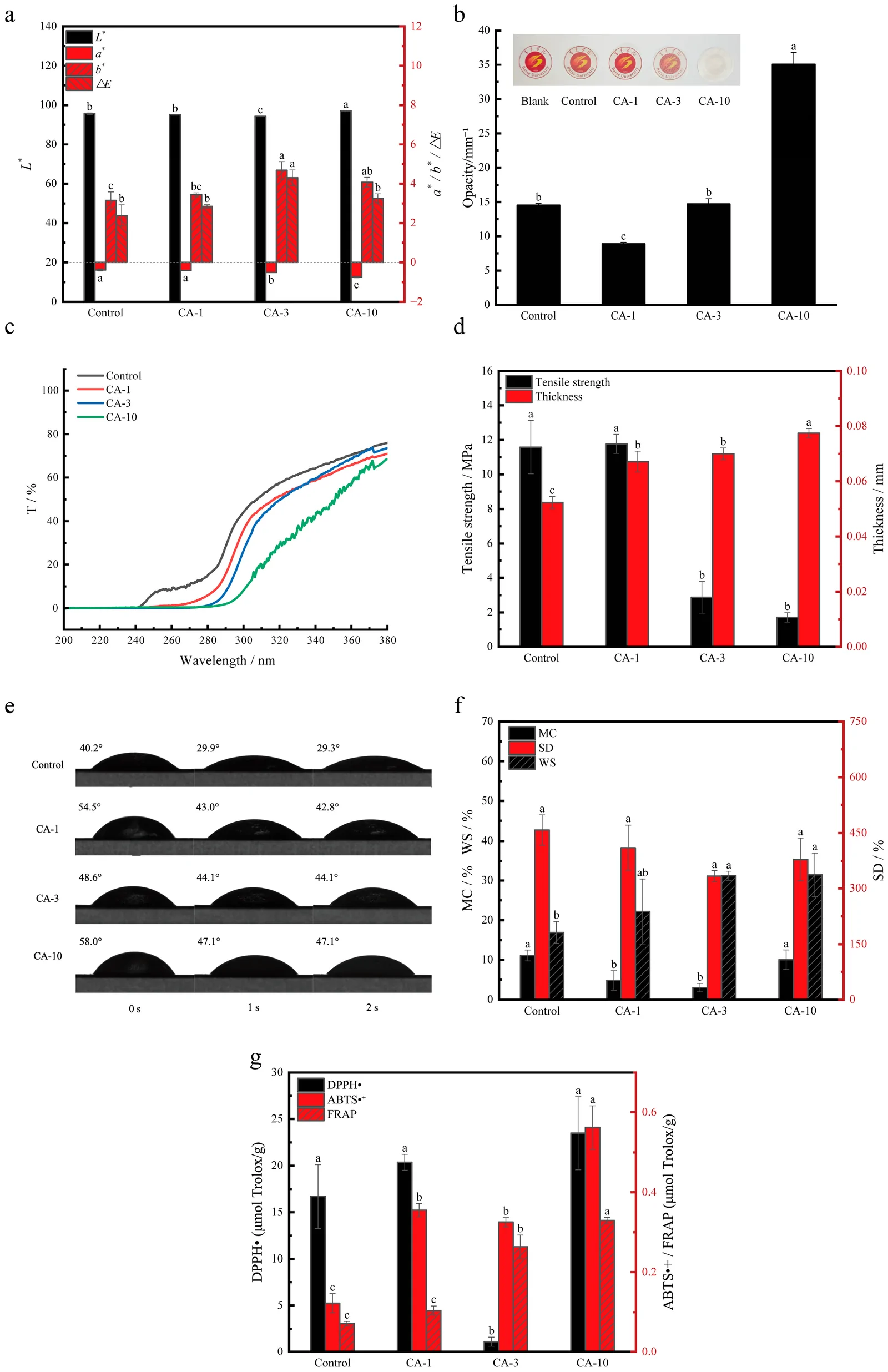
Dose-dependent physicochemical and functional responses of CA-modified films: (**a**) color parameters (L*, a*, b*, and ΔE); (**b**) opacity and visual transparency (inset images); (**c**) UV–Vis transmittance spectra; (**d**) tensile strength and thickness; (**e**) dynamic water contact angle images at 0, 1, and 2 s; (**f**) water sensitivity indicators (MC, SD, and WS); and (**g**) antioxidant activity (DPPH•, ABTS•^+^, and FRAP). Different lowercase letters indicate significant differences among groups (*p* < 0.05), and the same letter indicates no significant difference (*p* > 0.05). The contact-angle images in panel (**e**) are presented as descriptive observations and were not subjected to inferential statistical analysis.

Color parameters further reflected the effect of CA on film appearance (Figure 3a). The L* values of all groups remained generally high, indicating good overall brightness, and high-dose CA did not cause obvious darkening. In contrast, a* and b* were more sensitive to CA. The a* values of all groups were negative, indicating an overall greenish tone, and a* decreased further (became more negative) after CA addition, suggesting a shift toward greener coloration. Meanwhile, b* increased overall (especially in the CA-3 group), indicating enhanced yellowness. Changes in ΔE indicate that the effect of CA on total color difference was dose-dependent but not strictly monotonic. In addition to the intrinsic color contribution of CA, microstructure-related scattering may also contribute to the observed color differences, as structural heterogeneity and refractive-index discontinuities can alter light propagation in polymer films [28,29,30,31,32]. This interpretation is consistent with the XRD/SEM observations presented in Section 3.2, which showed changes in ordered domains and a more heterogeneous morphology at higher CA loadings.

The opacity results showed clear dose-dependent divergence (Figure 3b). Compared with the Control, low-dose CA (CA-1) reduced opacity, indicating improved visible transparency. However, as CA loading further increased (especially in CA-10), opacity increased significantly and film transparency decreased markedly. This trend was consistent with the SEM observations showing more frequent cross-sectional voids and greater interfacial nonuniformity in the high-dose group. Although a direct relationship between the observed void features and opacity was not quantitatively established in the present study, the observed structural heterogeneity may partly contribute to the increased opacity. Porous and heterogeneous polymer structures can create local refractive-index discontinuities, which enhance light scattering and reduce light transmission [28,29,30,31,32]. Such microstructure-induced scattering may also alter the apparent color of the films. Therefore, the observed optical changes at high CA loading may reflect a combination of CA-associated light absorption and microstructure-related light scattering [28,29,30,31,32,33]. Nevertheless, the SEM observations should be regarded as qualitative structural evidence supporting, rather than directly demonstrating, the observed changes in film opacity. Within the tested formulations, the optical response therefore differed with CA loading, with lower opacity at CA-1 but higher opacity at CA-10.

The UV–Vis transmittance results showed that the CA-containing films exhibited enhanced UV-shielding ability compared with the Control (Figure 3c). In the 200–380 nm range, the transmittance of the CA-treated groups was consistently lower than that of the Control, and decreased further with increasing CA loading; the CA-10 group exhibited the strongest UV-blocking effect. The lower UV transmittance of the CA-containing films is consistent with the UV-absorbing contribution of aromatic and conjugated structures reported for active biopolymer films [32,33]. In the present study, this interpretation is also consistent with the Raman responses at 765/850/1180 cm^−1^ presented in Section 3.1.2. Accordingly, the improved UV-shielding performance is interpreted as being associated with CA-related aromatic contributions, rather than as direct evidence of a specific molecular mechanism.

#### 3.3.2. Mechanical Integrity and Thickness

CA addition significantly affected the thickness and mechanical integrity of the composite films (Figure 3d). Overall, film thickness increased progressively with increasing CA loading (*p* < 0.05). However, because the total solids content of the film-forming solutions and the residual CA content in the dried films were not directly determined, the mechanism underlying this increase cannot be conclusively established. The observed thickness variation may reflect changes in the composition and structural organization of the film-forming matrix associated with CA incorporation [25,26]. In contrast to the increase in thickness, tensile strength showed a clear dose-dependent deterioration trend. No significant difference was observed between CA-1 and the Control (*p* > 0.05), indicating that the low CA loading did not substantially reduce tensile strength under the tested conditions. However, the tensile strength of CA-3 and CA-10 decreased significantly (*p* < 0.05), suggesting reduced mechanical integrity at higher CA loadings. These mechanical changes are consistent with the XRD and SEM results presented in Section 3.2, which showed weakened ordered domains together with more frequent voids and less compact cross-sectional regions at higher CA loadings. The combined observations suggest that the reduced tensile strength at higher CA levels may be associated with decreased structural continuity of the film matrix [34]. Importantly, the increased film thickness at CA-10 did not correspond to improved mechanical integrity. At high CA loading, excessive incorporation of the hydrophobic compound may interfere with intermolecular interactions within the Gel/KGM network and increase structural discontinuities, resulting in a thicker but mechanically weaker matrix [35,36]. Similar reductions in mechanical strength following high loading of hydrophobic active compounds have been reported in KGM- and gelatin-based composite films [37]. However, tensile strength alone does not provide a complete description of film stiffness and deformation behavior. Since elastic modulus and Poisson’s ratio were not determined in the present study, the mechanical integrity and resistance to failure of CA-10 under practical-use conditions cannot be conclusively established. Further studies incorporating these parameters and application-specific mechanical tests are therefore warranted.

#### 3.3.3. Surface Wettability and Water Sensitivity

Representative contact-angle observations are shown in Figure 3e. Because only one film and one droplet at one position were examined for each formulation, these measurements were interpreted descriptively rather than subjected to inferential statistical analysis. The initial contact angle (0 s) of the Control film was approximately 40.2° and decreased to 29.3° within 2 s, indicating rapid droplet spreading in this measurement. In comparison, the CA-containing films generally showed higher observed contact angles. In particular, the contact angle of CA-10 decreased from 58.0° at 0 s to 47.1° at 2 s, while CA-1 and CA-3 showed similar temporal trends. These observations are consistent with a tendency toward reduced surface wettability after CA incorporation. Considering the responses of the aromatic- and CH-related windows in FTIR/Raman, the hydrophobic structural units of CA may have contributed to changes in the surface chemical environment of the films [32]. However, additional measurements using independently prepared films are required to establish the statistical significance of this effect.

The effect of CA on the water sensitivity of the composite films exhibited a clear “surface–bulk divergence” pattern (Figure 3f). MC was lower in the CA-1 and CA-3 groups than in the Control, whereas it increased again in CA-10, indicating that low-to-medium CA loadings may have reduced the proportion of free or weakly bound water in the films, while the increase at the highest loading may be associated with the less compact cross-sectional regions observed by SEM in Section 3.2.2. In contrast, SD showed relatively small differences among groups, suggesting that CA had a limited effect on short-term swelling behavior under the conditions examined.

In contrast, WS showed an overall increasing trend with CA addition, and the CA-3 and CA-10 groups were significantly higher than the Control (*p* < 0.05). These results suggest that, at higher CA loadings, the reduced structural continuity indicated by the XRD and SEM observations presented in Section 3.2 may have facilitated water penetration into the bulk phase and the release of soluble components, thereby contributing to the higher WS.

These results indicate that water sensitivity cannot be interpreted from the contact-angle observations alone, because both surface wettability and bulk structural continuity may contribute to the overall response. Together with the FTIR/Raman, XRD, and SEM results presented in Section 3.1 and Section 3.2, the present observations provide a plausible structural interpretation for the tendency toward reduced surface wettability together with increased WS at higher CA loadings [38].

#### 3.3.4. Antioxidant Activity

CA addition affected the antioxidant responses measured by the three assays, with different response patterns observed for DPPH•, ABTS•^+^, and FRAP (Figure 3g). For ABTS^•+^ scavenging activity and FRAP reducing power, the CA-treated groups were overall higher than the Control and showed increasing trends with increasing CA loading, with CA-10 reaching the highest level (*p* < 0.05). These results indicate that CA addition enhanced the radical-scavenging and reducing capacities measured for the film extracts, although the residual CA content in the dried films was not directly quantified.

In contrast, DPPH^•^ scavenging activity exhibited a nonlinear dose response: low-dose CA (CA-1) showed little difference from the Control or a slight increase, the medium-dose CA-3 group decreased, and the high-dose CA-10 group increased significantly again. The observed variation in DPPH• scavenging activity may be associated with differences in CA dispersion, active-site accessibility, migration/release behavior, and film network characteristics [39].

It should be noted that DPPH^•^, ABTS^•+^, and FRAP reflect different antioxidant reaction pathways; therefore, their trends for the same sample are not necessarily consistent. Moreover, these assays differ in their reaction mechanisms, reaction environments, and reaction kinetics [40,41,42]. DPPH• scavenging can be particularly influenced by reaction kinetics, steric accessibility, and diffusion of antioxidants toward the DPPH radical [40], whereas ABTS•^+^ can react with a broader range of hydrophilic and lipophilic antioxidants. FRAP, in contrast, primarily reflects electron-donating capacity through the reduction of Fe^3+^ to Fe^2+^ rather than direct radical scavenging [42]. In particular, DPPH• usually shows weaker correlation with ABTS•^+^/FRAP and is more easily affected by sample microstructure and mass-transfer behavior [43].

Therefore, the decrease in DPPH• scavenging activity at CA-3 should not be attributed solely to active-site blocking. Rather, it may represent an assay-dependent response associated with the intermediate structural state of the film and the kinetic and mass-transfer constraints of the DPPH• reaction. Combined with the XRD and SEM results presented in Section 3.2, the structural rearrangement, more heterogeneous morphology, and more frequent voids observed at medium-to-high CA loadings may have influenced active-site exposure and mass-transfer processes, thereby contributing to the different response patterns among the antioxidant assays. The differences observed in film structure may partly explain the distinct DPPH• response without necessarily implying corresponding changes in ABTS•^+^ scavenging or ferric-reducing capacity. Overall, ABTS•^+^ and FRAP demonstrate the antioxidant enhancement associated with CA addition, whereas the variation in DPPH• scavenging activity may reflect differences in assay-specific reaction mechanisms and antioxidant accessibility.

### 3.4. Integrated Analysis of Structure and Performance Across CA Loadings

Based on the combined FTIR, Raman, XRD, SEM, and macroscopic performance results, the responses of Gel/KGM composite films can be interpreted in relation to the selected CA loading levels. It should also be noted that Tween-80 was fixed to maintain a consistent emulsification protocol across treatments. As CA loading increased, the emulsifier-to-CA ratio decreased accordingly, which may have contributed to differences in CA dispersion state, especially at higher loadings, and should therefore be considered when interpreting the observed dose-dependent responses.

At the low CA loading (CA-1), the spectroscopic results indicated changes in aromatic-, carbonyl-, and CH-related vibrational regions, whereas the XRD and SEM observations showed only limited changes in ordered domains and microstructure. At the macroscopic level, CA-1 showed a tendency toward reduced surface wettability, lower opacity, and maintained tensile strength relative to the Control, together with changes in antioxidant performance. Thus, the low CA loading was associated with relatively limited structural disturbance while retaining several functional benefits.

In the medium-dose stage (represented by CA-3), CA appears to exert stronger influence over molecular vibrations, local ordered domains, and morphological structure, as evidenced by more pronounced band reconstruction, ordered-domain disturbance, and surface/cross-sectional morphological rearrangement. At this loading, stronger UV-shielding and antioxidant responses were observed, while structural costs also became evident, including reduced tensile strength and increased WS. Therefore, among the tested formulations, CA-3 may represent a possible compromise between structural integrity and functional performance.

In the high-dose stage (represented by CA-10), CA-associated structural disturbance became more pronounced: FTIR/Raman showed more pronounced redistribution of vibrational contributions and greater local environmental heterogeneity, XRD showed marked weakening of ordered domains and reconstruction of peak clusters, and SEM showed more frequent cross-sectional voids, less compact regions, and a more heterogeneous surface morphology. Although UV blocking and antioxidant functions continued to improve macroscopically, these gains were accompanied by decreased tensile strength, increased water solubility, and reduced transparency, suggesting that high CA loading may shift the role of CA from a “structure-regulating factor” toward a “source of structural disturbance.” These observations indicate a trade-off between functional gains and structural stability across the tested formulations [44].

This structure–property interpretation provides a possible explanation for the observed responses of Gel/KGM composite films to different CA loadings and may serve as a reference for future formulation and mechanistic studies.

## 4. Conclusions

Using Gel/KGM composite films as the matrix and CA loading as the primary formulation variable, this study systematically evaluated the effects of CA on film structure and performance. The results showed CA-dependent changes in the local chemical environment, ordered domains, and microstructure of the composite films. FTIR and Raman spectroscopy revealed changes in carbonyl-, aromatic-, and C–H-related spectral regions, including variations in peak position, COG, and band shape, suggesting modifications in the local molecular environment following CA addition. XRD patterns qualitatively suggested weakening and rearrangement of ordered domains with increasing CA loading, while residual ordered domains remained detectable. SEM observations showed greater surface heterogeneity and more frequent voids and less compact regions in the cross-sections at higher CA levels. In terms of performance, representative contact-angle observations suggested a tendency toward reduced surface wettability after CA addition, while CA also improved UV-shielding ability and antioxidant activity; high CA loading, however, reduced mechanical integrity and increased water solubility. Overall, the results indicate a trade-off between functional enhancement and structural stability, with CA-3 representing a possible compromise between structural integrity and functional performance among the tested formulations. Future studies should examine a narrower CA loading range to determine whether the apparent compromise near CA-3 is reproducible and should evaluate candidate formulations using real food systems or appropriate food simulants under practical storage conditions. Additional barrier properties, including water vapor permeability (WVP), water vapor transmission rate (WVTR), and gas permeability, should also be evaluated.

## Figures and Tables

**Table 1:** Composition of Gel/KGM film-forming formulations with different cuminaldehyde (CA) levels.

Component	Control	CA-1	CA-3	CA-10
Gelatin (Gel), g	12.0	12.0	12.0	12.0
Konjac glucomannan (KGM), g	0.4	0.4	0.4	0.4
Glycerol, g	0.3	0.3	0.3	0.3
Ultrapure water, mL	100	100	100	100
Tween-80, mL	1	1	1	1
Cuminaldehyde (CA), mL	0	1	3	10
CA, mL/g polymer	0	0.081	0.242	0.806
CA, wt% of nominal total formulation	0	0.85	2.52	7.92
Film-forming solution used for casting, g	25	25	25	25

Gel, gelatin; KGM, konjac glucomannan; CA, cuminaldehyde. CA levels were expressed both as the amount added to each formulation, relative to the total polymer content (Gel + KGM, 12.4 g), and as wt% of the nominal total formulation. The latter was calculated using densities of 0.979 g/mL for CA and 1.06 g/mL for Tween-80, with the density of water taken as 1.00 g/mL. No further adjustment of the final mass or volume was performed after CA addition. For film casting, 25 g of each resulting film-forming solution was used.

**Table 2:** Stability and dose sensitivity of the FTIR and Raman reference bands.

Spectrum	FTIR	Raman
Band center/Integration range	1360 cm^−1^/1340–1380 cm^−1^	2889 cm^−1^/2840–2890 cm^−1^
peak position SD	0.000 cm^−1^	1.120 cm^−1^
CV, depth/height	0.210	0.093
CV, area	0.201	0.122
Spearman’s ρ (depth/height vs. dose)	0.4	0.6
Spearman’s ρ (area vs. dose)	0.4	0.6
Standardized slope, height	0.025	0.008
Standardized slope, area	0.026	0.011

Reference-band metrics were extracted from baseline-corrected spectra. For FTIR, peak depth and area were used; for Raman, peak height and area were used. Trend metrics (Spearman’s ρ, slope, and standardized slope) were calculated using the group means of the four CA loading levels (0, 1, 3, and 10 mL). The standardized slope was defined as the linear slope divided by the median of the corresponding metric across the four CA levels.

**Table 3:** Dose–response and band-shape parameter changes in characteristic FTIR spectral regions of films after normalization to the reference band at 1360 cm^−1^ (1340–1380 cm^−1^).

Band ID	Wavenumber Range (cm^−1^)	Area Ratio	Spearman’s ρ	Standardized Slope	CV	ΔPeak Position (cm^−1^)	ΔCOG (cm^−1^)	ΔFWHM (cm^−1^)
O–H/N–H stretching region	3100–3600	×1.003	0.6	0.002	0.029	−3.857	+3.547	0.579
Aliphatic C–H stretching region	2800–3000	×2.574	1.0	0.050	0.365	−6.991	−13.530	47.080
Carbonyl (C=O) band (~1734 cm^−1^)	1710–1760	×9.712	0.8	0.095	0.698	+24.106	+16.915	—
C=N/Amide I overlapping region (tentative assignment)	1640–1690	×1.079	1.0	0.008	0.037	0.000	+0.050	—
Amide I region	1600–1680	×1.079	1.0	0.008	0.037	+1.205	+0.001	—
Amide II region	1500–1585	×1.027	0.8	0.004	0.020	+0.482	−1.109	—
Amide III region	1200–1285	×1.020	0.8	0.001	0.009	+0.482	+0.190	−1.923
Polysaccharide C–O–C/C–O region	1050–1120	×2.168	0.8	0.040	0.321	+1.446	+6.099	—
Polysaccharide C–O region	1000–1050	×1.411	0.8	0.017	0.167	0.000	+0.111	—
Aromatic ring-related band (~848 cm^−1^)	820–870	×1.113	0.4	0.028	0.223	+27.963	+2.744	—

FTIR band metrics were extracted from baseline-corrected FTIR spectra after normalization to the reference band near 1360 cm^−1^ (1340–1380 cm^−1^). Area ratio was calculated as the ratio of the band area in the CA-10 group to that in the Control group. Trend metrics (Spearman’s ρ, slope, and standardized slope) were calculated using the group means of the four CA loading levels (0, 1, 3, and 10 mL) for the band-area response. The standardized slope was defined as the linear slope divided by the median band area across the four CA levels. CV denotes the coefficient of variation of band area. ΔPeak position, ΔCOG, and ΔFWHM are expressed as the difference between the CA-10 and Control groups. The 1640–1690 cm^−1^ window was evaluated as a composite C=N/Amide I overlapping region, whereas the 1600–1680 cm^−1^ window was analyzed separately as the conventional Amide I region. These partially overlapping windows were used for different analytical purposes and were not treated as independent spectral assignments.

**Table 4:** Dose–response and band-shape parameter changes in characteristic Raman spectral regions of films after normalization to the reference band at 2889 cm^−1^ (2840–2890 cm^−1^).

Band ID	Wavenumber Range (cm^−1^)	Area Ratio	Spearman’s ρ	Standardized Slope	CV	ΔPeak Position (cm^−1^)	ΔCOG (cm^−1^)	ΔFWHM (cm^−1^)
R765 (aromatic ring- related vibration)	740–790	×7.286	1.0	0.567	1.162	−23.724	−1.700	—
R850 (aromatic ring- related vibration)	830–875	×2.132	0.4	0.110	0.723	−6.850	−1.334	—
R925 (fingerprint-region band)	910–945	×1.761	0.4	0.057	0.671	1.941	0.525	—
R1025 (fingerprint- region band)	1005–1045	×1.793	0.2	0.201	1.001	−8.617	−1.843	—
R1095 (polysaccharide-related band)	1075–1110	×0.531	−0.4	−0.02	0.97	1.900	1.722	—
R1180 (aromatic/ coupled vibration band)	1155–1200	×8.775	0.4	0.099	0.903	−10.307	−7.969	—
R1245 (Amide III- related band)	1225–1265	×0.745	−0.8	−0.028	0.136	−7.457	−2.114	—
R1320 (Amide III/CH deformation-related band)	1300–1340	×0.774	−0.8	−0.012	0.531	−1.843	0.216	—
R1400 (CH_2_/CH_3_ deformation-related band)	1385–1420	×1.367	0.8	0.012	0.297	−18.223	−1.276	—
R1450 (CH_2_/CH_3_ deformation-related band)	1435–1475	×1.148	0.4	0.026	0.151	0.907	0.364	—
R1665 (Amide I/carbonyl-coupled region)	1645–1685	×1.329	0.2	0.048	0.247	3.530	0.600	—
R2935 (CH_2_/CH_3_ stretching-related band)	2895–2975	×0.855	−0.8	−0.025	0.16	−2.220	−1.225	0.838

Raman band metrics were extracted from corrected Raman spectra after normalization to the selected reference band near 2889 cm^−1^ (2840–2890 cm^−1^). Area ratio was calculated as the ratio of the band area in the CA-10 group to that in the Control group. Trend metrics (Spearman’s ρ, slope, and standardized slope) were calculated using the group means of the four CA loading levels (0, 1, 3, and 10 mL) for the band-area response. The standardized slope was defined as the linear slope divided by the median band area across the four CA levels. CV denotes the coefficient of variation of band area. ΔPeak position, ΔCOG, and ΔFWHM are expressed as the difference between the CA-10 and Control groups.

## Data Availability

The original contributions presented in this study are included in the article. Further inquiries can be directed to the corresponding author.
